# Etiology and Management of Raised Intraocular Pressure following Posterior Chamber Phakic Intraocular Lens Implantation in Myopic Eyes

**DOI:** 10.1371/journal.pone.0165469

**Published:** 2016-11-17

**Authors:** Sirisha Senthil, Nikhil S. Choudhari, Pravin K. Vaddavalli, Somasheila Murthy, Jagadesh Reddy, Chandra S. Garudadri

**Affiliations:** 1 VST Glaucoma Center, L V Prasad Eye Institute, Banjara Hills, Hyderabad, India; 2 Tej Kohli Cornea Services, L V Prasad Eye Institute, Banjara Hills, Hyderabad, India; University of Illinois at Chicago, UNITED STATES

## Abstract

**Aim:**

To evaluate the etiology and management of elevated intraocular pressure (IOP) following posterior chamber phakic implantable collamer lens (ICL) surgery.

**Methods:**

Between 2009 and 2015, 638 eyes of 359 subjects with refractive myopia, underwent V4b and V4c (CentraFLOW) model ICL implantation. Ocular hypertension (OHT) was defined as IOP of ≥ 22 mm Hg on two separate occasions and elevated IOP with corresponding optic disc or visual field damage was defined as glaucoma.

**Results:**

Elevated IOP ≥ 22 mm Hg was noted in 33 eyes of 30 subjects (33/638; 5.17%). Median age of subjects with raised IOP was 26 years (Inter quartile range (IQR):22, 29) and median refarctive error was -16 diopters (-19.5, -13). The median follow up was 7.8 months (IQR:0.3, 17.6) and median time for postoperative IOP rise was 12 days, (IQR:2, 24). The various etiologies for elevated IOP were steroid response in 21 eyes (64%; 10 eyes with V4b, 11 eyes with V4c), retained viscoelastic in 5 eyes (15%) (3 with V4b, 2 with V4c), pupillary block in four eyes (12%; 3 with V4b, 1 with V4c), malignant glaucoma in one eye (3%, V4b), and missed pre-existing Juvenile open angle glaucoma (JOAG) in two eyes (6% with V4b). Elevated IOP in 31 eyes resolved with conservative management. One eye (centraFLOW design) with central aquaport block by viscoelastic, needed AC wash and one eye with malignant glaucoma needed parsplana vitrectomy and hyaloidotomy. Ten eyes required longterm (>2 months) antiglaucoma medications (AGM) for IOP control. Except the two eyes with JOAG, none had disc and field damage.

**Conclusion:**

In our series, OHT was seen in 4.85% and glaucoma in 0.3% eyes that underwent V4b and V4c model ICL implantation. Multiple etiologies were noted and steroid induced ocular hypertension was the most common cause of elevated IOP followed by retained viscoelastic and pupillary block. One third of these eyes required longterm AGM for IOP control.

## Introduction

Phakic posterior chamber intraocular lens (PIOL) implantation is a widely accepted and performed refractive surgery for correction of high myopia, when corneal laser ablative procedures are not suitable. PIOLs have shown promising long term results in terms of faster visual recovery, high efficacy and reversibility [1–2]. Although considered a safe procedure, PIOL implantation may be associated with complications such as cataract formation, post-operative intraocular pressure (IOP) elevation and endothelial cell loss [3].

The Visian Implantable Collamer Lens (ICL) (STAAR surgical, Nidau, Switzerland) is a commonly used posterior chamber, ciliary sulcus placed ICL. It is a single piece collamer lens with a plate haptic design and anterior lens vaulting [1–2]. The anterior lens vaulting, designed to prevent crystalline lens contact, predisposes the eye to pupillary block glaucoma. To prevent pupillary block, prophylactic peripheral iridotomy (PI) is recommended with the V4b model of the lens [1,4]. Two laser PIs are recommended less than 180 degrees apart, the second one as a safeguard in case the ICL rotates and the haptic blocks the PI [2]. The new V4c model of ICL (CentraFLOW) has a 0.36mm central aperture (Aquaport) in the optic, designed to allow aqueous flow and hence prevent pupillary block. Hence peripheral iridotomy is not advocated with these lenses [5].

Raised IOP following ICL implantation can be seen in the early or late post operative period and can be due to open angle or angle closure mechanisms [2, 6–18]. The rate of occurrence of raised IOP as repoted in literature ranges from 0.8% to 26.2% [3, 19]. Understanding the mechanisms of postoperative raised IOP is important to plan appropriate treatment and prevent longterm sight threatening complications of glaucoma. The aim of our study was to look at the various etiologies for raised IOP following V4b and V4c model phakic ICL implantation, to discuss their management and outcomes in a consecutive case series.

## Materials and Methods

The medical records of 359 consecutive subjects (638 eyes) who underwent posterior chamber implantable collamer lens for high myopia, between June 2009 and July 2015 were reviewed. The study protocol was approved by the Ethics committee of L V Prasad Eye Institute, (EC ref No: LEC 01-16-017). The written informed consent was given by participants for their clinical records to be used in this study. Cornea and refractive surgeons performed the ICL surgeries and the surgical protocols were similar. An IOP ≥ 22 mmHg (recorded by Goldmann Applanation tonometry) on two separate occasions was defined as ocular hypertension and IOP ≥ 22 mmHg with glaucomatous disc or field changes was defined as glaucoma.

Pre-operative evaluation included systemic and ocular history, refractive status, slit-lamp biomicroscopy, endothelial cell count, biometry, topographic keratometry, pachymetry, horizontal white-to-white corneal diameter using Orbscan Iiz (average of 3 measurements as recommended), intra ocular pressure, gonioscopy and detailed disc and retinal evaluation. All V4b model ICLs had two YAG PIs performed atleast a week prior to ICL procedure and no YAG PI for V4c model ICL. The manufacturer-advised ICL power (STAAR surgical, Nidau, Switzerland) was used for implantation under topical anesthesia. A 3.0-mm temporal clear corneal incision and 2 paracenteses were created, anterior chamber was filled with ocular viscoelastic device (OVD), we used Hydroxypropyl methyl cellulose (HPMC 2% w/v, Milmet, Sun Pharmaceuticals Industries Ltd, India). The ICL was then injected into the anterior chamber and the footplates was gently tucked beneath the iris. The viscoelastic was washed using automated coaxial irrigation and aspiration with balanced salt solution (BSS), pupil was constricted with intracameral pilocarpine (0.13 mg/ml pilocarpine in BSS). Postoperatively, topical prednisolone acetate 1% eye drop in tapering doses over 4 weeks and topical moxifloxacin 0.5% eye drops 4 times a day for 1 week were prescribed. Patients were followed up on day 1, 1 week, 1 month and 4–6 monthly there after. In case of an emergency or in the presence of a complication or elevated IOP, they were reviewed more often.

### Statistical analysis

Statistical analysis consisted of descriptive analysis. Continuous variables were summarized as mean and standard deviation if they were normally distributed and as median and interquartile range if non-normally distributed. Categorical variables were summarized as percentages. Chi square test was used to compare the proportions of glaucoma in the two groups. Statistical analysis was performed using commercial software (Stata ver. 11.2; StataCorp, College Station, Tx).

## Results

During the study period, 443 consecutive eyes (252 subjects) underwent V4b model from June 2009 untill July 2014 and 195 consecutive eyes (107 subjects) underwent V4c model ICL implantation from mid 2014 till july 2015 for correction of high myopia. Rise in IOP ≥22 mm Hg was noted in 19 of 443 eyes (4.28%) with V4b and 14 of 195 (7.17%) following V4c model ICL implantation. The difference in the prevalence of glaucoma between the two groups (2.89%) was was not significant (Chi ^2^ test, p = 0.076). The demographics and clinical charecteristics of subjects with raised IOP with both the models of ICL are given in Table 1. The preoperative and postoperative BCVA, preoperative IOP and last follow up IOP, HWW diameter were similar between the two groups. The ACD in all the eyes was greater than or equal to 2.75 mm. The median ACD similar in the two groups (ranksum: p = 0.86).The preoperative median myopic refractive error was significantly higher in the V4b model (p<0.01). All eyes had normal preoperative IOP. Seventeen of the 19 eyes with V4b model had YAG PI one week prior to ICL and the remaining two, had surgical iridectomy during the ICL implantation. None of the eyes with V4c model had any iridotomy.

**Table 1:** Demographics and preoperative characteristics of subjects with raised IOP following V4 b (n = 19) and V4c ICL (n = 14) implantation.

Parameter	V4b ICL, Median (Interquartile range)*	V4c ICL, Median (Interquartile range)
Age (years)	28 (22,30)*	23 (22, 28)
Right: Left	15:04	9:05
Male: Female	8:8	8:6
Spherical refractive error	-17 (-21, -15)*	-13.5 (-16, -11)
Preoperative BCVA	0.3 (0.1, 0.3)*	0.2 (0.2, 0.3)
Preoperative IOP	14 (13, 16)*	14.5 (14, 16)
HWW diameter	11.6 (11.2, 12.2)*	11.5 (11.4, 12)
Preoperative ACD	3.2 (2.82, 3.27)*	2.98 (2.9, 3.24)
Preoperative CDR	0.3 (0.3, 0.4)*	0.3 (0.3, 0.4)
**Postoperative characteristics**		
Time to IOP rise (days)	15 (1, 24)*	10 (4, 30)
Postoperative highest IOP	32 (26, 38)*	27.5 (25, 30)
IOP at final follow up	16 (12,17)*	14.5 (14, 18)
**Gonioscopy (postoperative)**		
Open angles	15 (3 eyes had increased TM pigment)	13 open angles
Closed angles	4 eyes, 3 with pupillary block and one with malignant glaucoma	1 closed angle
Postoperative BCVA	0.1 (0, 0.2)*	0.05 (0, 0.2)
Follow up (months)	16.9 (10.4, 25.1)*	1.25 (0.3, 1.8)
No of eyes that needed AGM at last FU	7	3
**AGM at last FU (number of eyes)**		
1 AGM	3	3
2 AGM	4	

n: number of subjects; BCVA: Best corrected visual acuity; IOP: intraocular pressure; AGM: Anti-glaucoma medication; FU: Follow up; PI: peripheral iridotomy; YAG: Yttrium Aluminum Garnet; CDR: Cup disc ratio; ACD: Anterior chamber depth; CCT: Central corneal thickness; HWW: Horizontal white to white; IQR: inter quartile range; TM: trabecular meshwork

*represents median with interquarter ranges

### Clinical evaluation of glaucoma

The rise in IOP postoperatively was observed between day 1 and day 42 after surgery, the median being 12 days (IQR: 2, 24). Preoperatively, all the eyes in the cohort had open angles. The postoperative gonioscopy showed closed angle in 4 of the 19 in eyes with V4b model ICL, three of the remaining 15 eyes eyes with open angles had excess pigmentation of posterior trabecular meshwork. Among eyes with raised IOP following V4c model ICL, 13 eyes had open angles and one eye had angle closure. The clinical details, types of glaucoma and their management, are summarised in S1 **Table**. Two of the 33 eyes had glaucomatous disc damage with corresponding visual field defect and all other eyes had normal optic disc.

### Possible mechanisms of raised IOP in our cohort

#### Retained viscoelastic or intraoperative AC overfill

Raised IOP in 5 eyes (15% (95% confidence intervals, (CI), 2.82–27.18); 3 eyes with V4b, 2 eyes with V4c) on postoperative day one, was attributed to retained viscoelastic or intraoperative anterior chamber overfill. These eyes responded to conservative management with oral and topical antiglaucoma medications. Three eyes resolved in 2 weeks and two of the eyes with persistent IOP elevation needed longterm antiglaucoma medication (AGM) for IOP control.

#### Steroid response

Twenty one eyes (63.6% (95% CI: 47.18–80.02); 10 eyes with V4b, 11 eyes with V4c) were diagnosed to have steroid induced ocular hypertension. Three of these eyes presented in the first week (although early for a steroid response, the contralateral eye also showed elevated IOP which subsided after shifting to low potent steroid) and the others presented between 2–4 weeks after surgery. These eyes were differentiated from overfill or retained viscoelastic. In eyes with steroid response, the IOP was under control in the immediate postoperative period and subsequently increased during follow up, in the absence of excess intraocular inflammation. The steroid responsiveness was managed either by stopping topical Prednisolone eye drops (if they presented at 3^rd^ or 4^th^ postoperative week) or switching to low potent steroid (if they presented at postoperative 1^st^ or 2^nd^ week,) and adding antiglaucoma medications as needed. Five out of 21 eyes required one longterm AGM (beyond 2 months post ICL surgery).

#### Pupillary block

**Fig 1:**
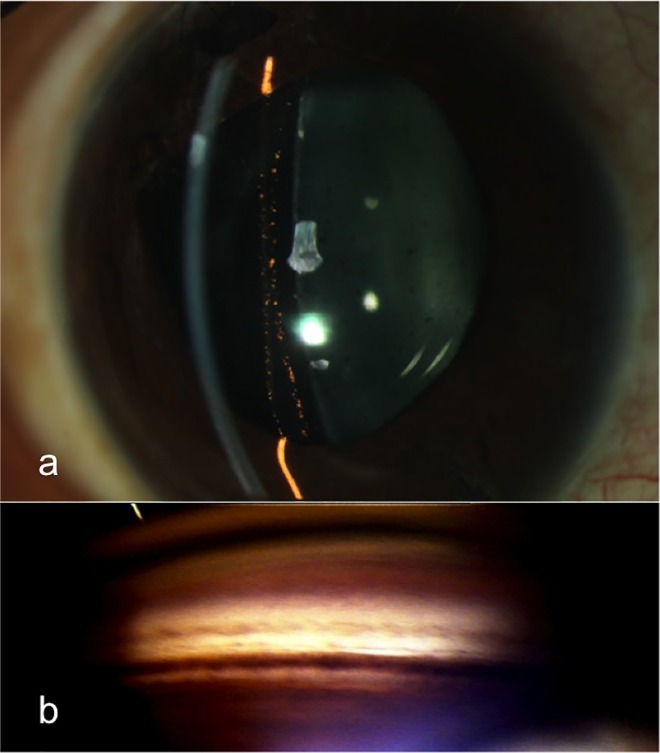
a) Slitlamp photograph with slit view showing pigment deposition on the ICL. b) Gonio photograph showing excess trabecular meshwork pigmentation.

Four eyes had pupillary block (12% (95% CI: 0.91–23.09); 3 with V4b, 1 with V4c). Three eyes with V4b model ICL had angle closure due to pupillary block. Two of these eyes had small YAG PIs, blocked with pigment debris and needed enlargement of the iridotomies, following that the AC deepened and the glaucoma resolved. In one of these eyes, the IOP remained controlled. However, the other eye required long-term AGM to control IOP, excess ICL vault and oversized ICL were ruled out by ASOCT (the vault was 370 microns0.37 mm). Possible excess pigment released during laser iridotomy was implicated as the likely cause for the prolonged rise in IOP (**Fig 1A and 1B**).

The third eye with two patent iridotomies had a pupillary block due to ICL rotation as the PIs were blocked by the ICL haptic and needed surgical iridectomy, following which the IOP was controlled. Non-pupillary block angle closure due to excess ICL vaulting was ruled out in this case since the measured ICL vault by ASOCT was 0.61 mm.

**Fig 2:**
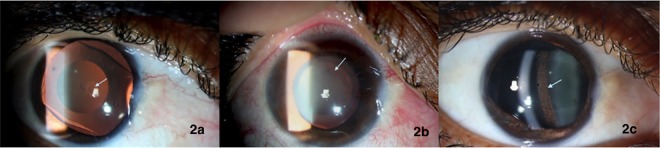
a): Slit lamp photograph showing microcystic corneal edema, fixed dilated pupil and viscoelastic behind the ICL seen on retroillumination. b): Slit lamp photograph with crystalline lens showing localized anterior capsular cataract. on retroillumination. c): One year post-operatively, pigments dispersed over the ICL surface and patent central aquaport.

One eye with V4c model ICL had acute pupillary block on postoperative day 1, presenting with a shallow anterior chamber and forward ICL shift. There was diffuse microcystic corneal edema with fixed, dilated pupil and an IOP of >60 mm Hg. After treatment with intravenous 20% Mannitol injection, there was resolution of corneal edema and it was noted that the central aquaport of the ICL was blocked with viscoelastic and inflammatory debris (**Fig 2A**). AC wash was performed on the same day and the residual viscoelastic in the AC and behind the ICL was thoroughly washed. The pupil failed to constrict intra-operatively even with intracameral pilocarpine. On day 2, the IOP was 14mm Hg with out any AGM. The pupil was fixed and dilated with pupillary sphincter atrophy. Pigment dispersion was noted on the anterior surface of the ICL. Crystalline lens showed a localized anterior capsular cataract (**Fig 2B**). One month post-operatively, the BCVA was 20/30 and IOP was 10 mm Hg without any anti glaucoma medications. Gonioscopy revealed open angles in all quadrants. The visual acuity and IOP control were well maintained at one-year follow up. However, the pupil remained fixed and dilated with pigments on the ICL surface (**Fig 2C).**

#### Preexisting juvenile open angle glaucoma (JOAG)

**Fig 3:**
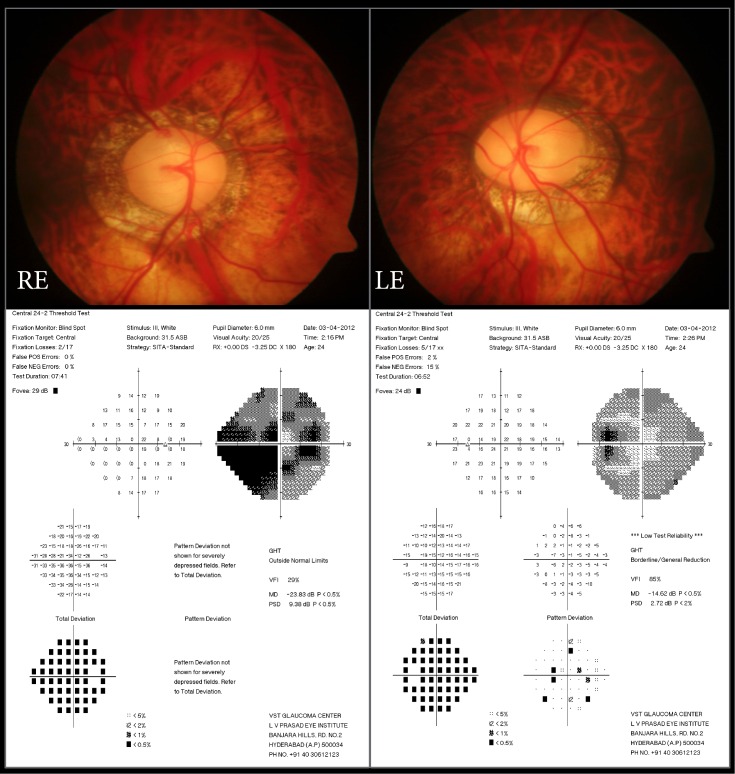
Optic disc photographs of the right eye (RE) and the left eye (LE) of the subject with JOAG and high myopia. Note the large discs with large cupping and neuroretinal rim thinning in both the eyes and the corresponding visual field changes.

One patient with V4b model of ICL had persistant high IOP postoperatively in both his eyes (operated 1 week apart). The IOP was 22 mm Hg and 31 mm Hg in the right and left eye respectively. Optic disc evaluation of the right eye showed advanced glaucomatous damage (0.9 cup disc ratio, CDR with bipolar notch) and the left eye showed early damage (0.8 CDR with thinning of the inferior neuroretinal rim with focal notch and a thin superior rim) (**Fig 3)** with corresponding visual field loss. Missed preexisting open angle glaucoma was the most likely cause and he was diagnosed as JOAG. This patient required two topical AGM in each eye for IOP control until last follow up (3 years post surgery).

#### Malignant glaucoma

**Fig 4:**
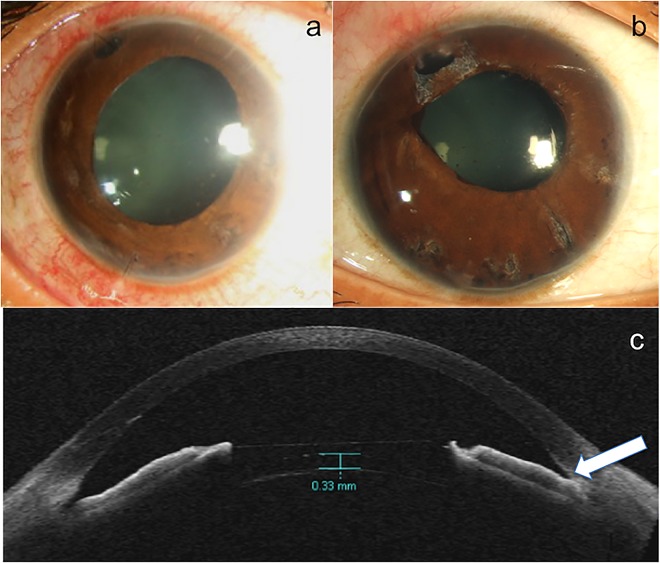
a) Slitlamp photograph of the eye with ICL and malignant glaucoma showing, patent surgical PI, dilated pupil and corneal edema. b) The eye with resolved malignant glaucoma with clear cornea, mid dilated pupil and iris atrophic patches following parsplana vitrectomy and hyaloidotomy. c) ASOCT of the same eye showing absence of excess ICL vaulting, deep anterior chamber with closed angle in one quadrant (white arrow) following pars plana vitrectomy.

One patient with V4b model ICL presented with an IOP of 45 mm Hg, shallow central and peripheral AC, patent peripheral iridotomy, four days post ICL surgery. Pupillary block was excluded, as there was a patent iridotomy; excess ICL vaulting was ruled out by ASOCT (the vault was 0.33 mm) and suprachoroidal hemorrhage was ruled out with ultrasound B scan, thus confirming the diagnosis of malignant glaucoma. This eye required parsplana vitrectomy and peripheral irido-zonulo-hyaloido-vitrectomy along with AC reformation. This eye had pupillary sphincter atrophy with dilated fixed pupil (possibly due to the acute rise in IOP) and segmental angle closure requiring 2 topical AGM for IOP control at final follow up of 6 months (**Fig 4**).

At final follow up, 10 eyes needed antiglaucoma medications; 3 eyes each with V4b and V4c model ICL needed one AGM for IOP control and 4 eyes with V4b model ICL needed 2 AGMs each for IOP control. Except for the two eyes that had pre-existing glaucoma, none of the others developed any glaucomatous disc damage or vision loss during the follow up period.

## Discussion

Posterior chamber phakic ICLs are increasingly used for refractive correction of high myopia [3]. Postoperative rise in IOP is reported in 0.8–26.2% of these eyes and multiple mechanisms have been implicated [3,19]. Some of the factors that predispose these eyes to glaucoma are: the position of the ICL in the ciliary sulcus that increases the risk of non-pupillary block angle closure and pigment dispersion, and the design of the ICL with anterior vaulting that predisposes the eye to pupillary block. Also, these eyes with high myopia are at a higher risk of steroid induced ocular hypertension and primary open angle glaucoma [20].

The early postoperative rise in intraocular pressure following ICL implantation is most often transient and is managed conservatively [9, 11–12]. Although less frequent, prolonged rise in IOP needing longterm AGM and/or surgical intervention have also been reported [6, 15, 17]. Understanding the mechanisms of postoperative elevated IOP is important to plan appropriate treatment and prevent longterm sight threatening complications of glaucoma.

In our series, 33 eyes out of 638 eyes (5.17% eyes) had elevated IOP following ICL implantation for high myopia. The commonest cause of elevated IOP in our series was steroid response (63.6% eyes), followed by retained viscoelastic (15%) and pupillary block (12%). There was transient elevation of IOP in 23 eyes (70%), which could be controlled after appropriate treatment. Ten eyes (30%) needed long-term AGM for IOP control and three eyes needed surgical intervention to control the IOP. In none of the eyes needed ICL explantation.

Steroid induced IOP rise is reported as one of the commonest causes of elevated IOP following ICL implantation [3,7], occurring within 1–4 weeks [8]. In our series also, the commonest cause of elevated IOP was steroid induced ocular hypertension (63.6% eyes), and the elevated IOP was noted within 1 week in 3 eyes and between 2–4 weeks in the remaining 18 eyes. The steroid response was more commonly seen in eyes with V4c model compared to V4b model (10/19 versus 11/14 eyes). This cannot be attributed to the ICL model, as the postoperative steroid regime was similar between the two groups. It is possible that incidentally, more number of eyes that underwent the V4c model ICL were steroid responders. The steroid response may not lead to glaucoma if diagnosed early and intervened appropriately. If IOP remains elevated for prolonged period, glaucomatous optic nerve damage can occur [2, 21].

Following this review we have changed our practice pattern and we currently use prednisolone acetate 1% eye drops only for the first week followed by Loteprednol 0.5% in tapering dose over 3–4 weeks. In 16 of the 21 eyes (75% eyes with steroid response) stopping the steroid eye drops along with short course of AGM helped to control the IOP. The remaining 5 eyes required AGM beyond 2 months to control the IOP. Prolonged ocular hypertensive response is reported in a small percentage of eyes with steroid response requiring longterm AGM [19].

Retained viscoelastic or overfill of the anterior chamber presents in the immediate postoperative period and often resolves with short course of antiglaucoma and anti-inflammatory medications. Elevated IOP due to retained viscoelastic is more common with high viscous ocular viscoelastic devices (OVD) like Sodium hyaluronate compared to hydroxyl propyl methyl cellulose (HPMC) [9,19]. In a series by Almalki et al, retained OVD was responsible for elevated IOP in majority of the cases (39.7%) followed by steroid response (37.9% eyes) [19]. Irrigating out the viscoelastic instead of irrigation and aspiration was one of the reasons for increased incidence of retained viscoelastic and high IOP in their series, the IOP normalized with short term AGM and none required long term AGM for IOP control [19]. We used 2% HPMC in all our cases and OVD was removed using coaxial irrigation and aspiration. Despite this, we had 5 eyes with raised IOP due to retained OVD. Four of the 5 eyes resolved within one week with AGM and one eye required long term AGM (until 7 months) for IOP control.

ICL induced angle closure can be caused by pupillary block, nonpupillary block or a combination of both mechanisms. Absence of iridotomy, a small PI or an imperforate PI can cause pupillary block in eyes with V4b model of posterior chamber ICL [8,11,14,19, 22]. Rarely, despite a patent PI, pupillary block can ensue if the plate-haptics of the ICL block the PI due to ICL rotation [22]. This could happen if PIs are placed 180 degrees apart. In such cases, an additional iridotomy or a surgical iridectomy would be necessary. In our series, 3 eyes with V4b had pupillary block. The pupillary block was relieved following PI in all three eyes. Two eyes resolved with temporary AGM and one eye with excess TM pigmentation (after repeat PI) needed long term AGM for IOP control.

With the new V4c model of ICL (CentraFLOW), though transient IOP rise in the early postoperative period is reported [18], pupillary block is not reported with these lenses. In our series majority (10/13 eyes, 77%) had transient rise in IOP. One eye had pupillary block with very high IOP on day one, due to central aquaport block by the retained viscoelastic behind the ICL. This eye needed surgical intervention to clear the viscoelastic. This relieved the condition and IOP was well controlled after this without any AGM.

Pigment dispersion with excess TM pigmentation is a common occurrence following prophylactic laser iridotomy performed before ICL implantation of V4b model [8,9], This could be due to constant friction between the posterior iris surface and the ICL, in the presence of excess ICL vaulting [3,11], or due to anatomical alterations of the ciliary sulcus in the absence of excess ICL vaulting [9]. The increase in IOP if present is usually managed with AGM [9], and in recalcitrant cases, ICL explantation may be needed [12,19]. Increased TM pigmentation was seen in 3 eyes in our series in eyes with V4b ICL. The source of pigment release was laser iridotomy and/ or excess ICL vault (in these 3 eyes the ASOCT showed vault ranging from 0.37 to 0.75 mm, hence excess vault was ruled out. Two of these three eyes with excess TM pigmentation needed long term AGM to control the IOP. In a series by Chung et al [9], one out of 48 eyes with high IOP at postoperative one-week (due to increased TM pigmentation) needed long-term AGM (3 AGM at 4 years).

The nonpupillary block mechanism causing angle closure can happen due to oversized ICL [19]. Oversized ICL causes excess vaulting (>1 mm or 1000μ) [23], angle crowding and predisposes to angle closure. This can be confirmed on ASOCT. We did not encounter any eye with excess ICL vaulting and elevated IOP. We had one eye with ICL vault >1000 μ, however the angles were open and there was no pigment dispersion in that eye. The high IOP in this eye was related to steroid response, which subsided after stopping topical steroid and IOP was controlled with no AGM.

Malignant glaucoma or aqueous misdirection has been reported following ICL implantation in both hyperopic [24] and myopic eyes [19, 25]. The diagnosis of malignant glaucoma is made in the absence of pupillary block and excess ICL vaulting and the ultrasound B-scan has ruled out suprachoroidal hemorrhage. This condition is treated with strong cycloplegics, topical anti-inflammatory medications, aqueous suppressants and hyperosmotic agents [25]. If conservative management does not help, PPV with irido-hyaloidotomy with or without ICL explantation may be needed [25, 26]. Kodjikian. L et al, postulated that the smaller sized ICL in their case was responsible for development of malignant glaucoma. In our case, ciliary body irritation and inflammation by the sulcus placed ICL could have lead to anterior rotation of the ciliary process, shallowing of the AC and aqueous misdirection into the vitreous cavity. Medical treatment was not successful and PPV with irido-hyaloidotomy was needed to resolve the aqueous misdirection. The longterm requirement of AGM, in this eye was attributed to the synechial angle closure that developed as a result of forward shift of lens-ICL-Iris complex.

We had one patient in this series in whom a preexisting JOAG was missed. Evaluation of glaucoma in myopic discs is a challenge, due to the large disc size and oblique optic nerve insertion [27]. Also, peripapillay and posterior pole myopic changes of the retina could lead to visual field defects. Though difficult, an attempt should be made to differentiate these from glaucomatous field changes by disc and field correlation. Careful preoperative evaluation including stereoscopic disc evaluation and visual field testing could help to diagnose preexisting glaucoma in these eyes. Also in the presence of suspicious discs, a baseline visual field test is recommended to help compare future field tests (in case of suspicion of glaucoma at a later date). A baseline visual field could have possibly helped pick up the JOAG prior to ICL implantation in our patient.

Based on our experience from this series, we present a simple step-ladder approach to recognize the possible causes and mechanisms of raised IOP in myopic eyes after ICL implantation in S1 **Fig** Depending on the time of presentation, anterior chamber angle changes, ICL vaulting and optic disc evaluation, the various mechanisms can be identified and treated appropriately. It is very important to recognize and treat elevated IOP in these eyes to prevent long-term sight threatening complications of glaucoma in these young subjects who undergo refractive surgery.

Some of the limitations of our study are its retrospective nature, different follow up periods for the two ICL models and non availability of postoperative ASOCT measurement for all the eyes. The follow up period was longer for V4b model ICL (since that was available earlier) compared to V4c model. This is unlikely to infleunce the incidence of elevated IOP with the newer V4c model, since the commonest cause was steroid response and this occurs in the early postoperative period during topical steroid use.

In our series, IOP ≥ 22 mm Hg following ICL implantation was seen in 5.17% of eyes, OHT was seen in 4.85% eyes and glaucoma in 0.3% eyes. Of the various mechanisms for elevated IOP following ICL surgery, the most common cause was steroid response followed by retained viscoelastic and pupillary block. With the newer ICL model (centraFLOW) the incomplete PI, pupillary block and excess trabecular pigmentation post PI causing raise in IOP can be avoided. However, the other causes of elevated IOP would still be a problem. We recommend using low potent topical steroid eye drops in tapering doses in the postoperative period to prevent steroid induced ocular hypertension. A thorough removal of viscoelastic is necessary to prevent immediate postoperative elevated IOP. It is suggested to rule out JOAG by careful disc evaluation and visual field testing in suspicious optic discs. Glaucoma, following ICL implantation, although uncommon, can be vision threatening. It is important to understand the various causes of elevated IOP and plan their management appropriately.

## Supporting Information

S1 Fig Showing a step ladder approach to diagnose the possible mechanism of glaucoma post ICL implantation. Legends: ICL: Implantable collamer lens, IOP: Intraocular pressure, JOAG: Juvenile open angle glaucoma, PI: Peripheral iridotomy, TM: Trabecular meshwork.
(PDF)

S1 Table Showing the clinical details, the mechanism of glaucoma and their management (n = 33) (uploaded as supporting information). Legends: M:male; F:Female; N:No; Y:Yes, PI: peripheral iridotomy; REF: refractive error; ACD: Anterior chamber depth; OVD: Ocular viscoelastic device; ST: Steroid induced glaucoma; PB: pupillary block; PM: excess trabecular meshwork pigmentation; HWW:Horizontal white-to-white; ICL: Implantable collamer lens; BCVA: best corrected visual acuity, IOP: Intraocular pressure; AGM: Antiglaucoma medications; PPV: Pars Plana vitrectomy; CDR: cup to disc ratio; FU: follow up.
(XLSX)
