# Correction: Etiology and Management of Raised Intraocular Pressure following Posterior Chamber Phakic Intraocular Lens Implantation in Myopic Eyes

**DOI:** 10.1371/journal.pone.0172929

**Published:** 2017-02-22

**Authors:** Sirisha Senthil, Nikhil S. Choudhari, Pravin K. Vaddavalli, Somasheila Murthy, Jagadesh C. Reddy, Chandra S. Garudadri

The fifth author’s name is incorrect in the published article. The correct name is: Jagadesh C. Reddy. The correct citation is: Senthil S, Choudhari NS, Vaddavalli PK, Murthy S, Reddy JC, Garudadri CS (2016) Etiology and Management of Raised Intraocular Pressure following Posterior Chamber Phakic Intraocular Lens Implantation in Myopic Eyes. PLoS ONE 11(11): e0165469. doi:10.1371/journal.pone.0165469
