# Supplementary figures and images for: Etiology and Management of Raised Intraocular Pressure following Posterior Chamber Phakic Intraocular Lens Implantation in Myopic Eyes
Source: PLoS One. 2016 Nov 17;11(11):e0165469. doi: 10.1371/journal.pone.0165469 (PMC5113895; doi:10.1371/journal.pone.0165469)

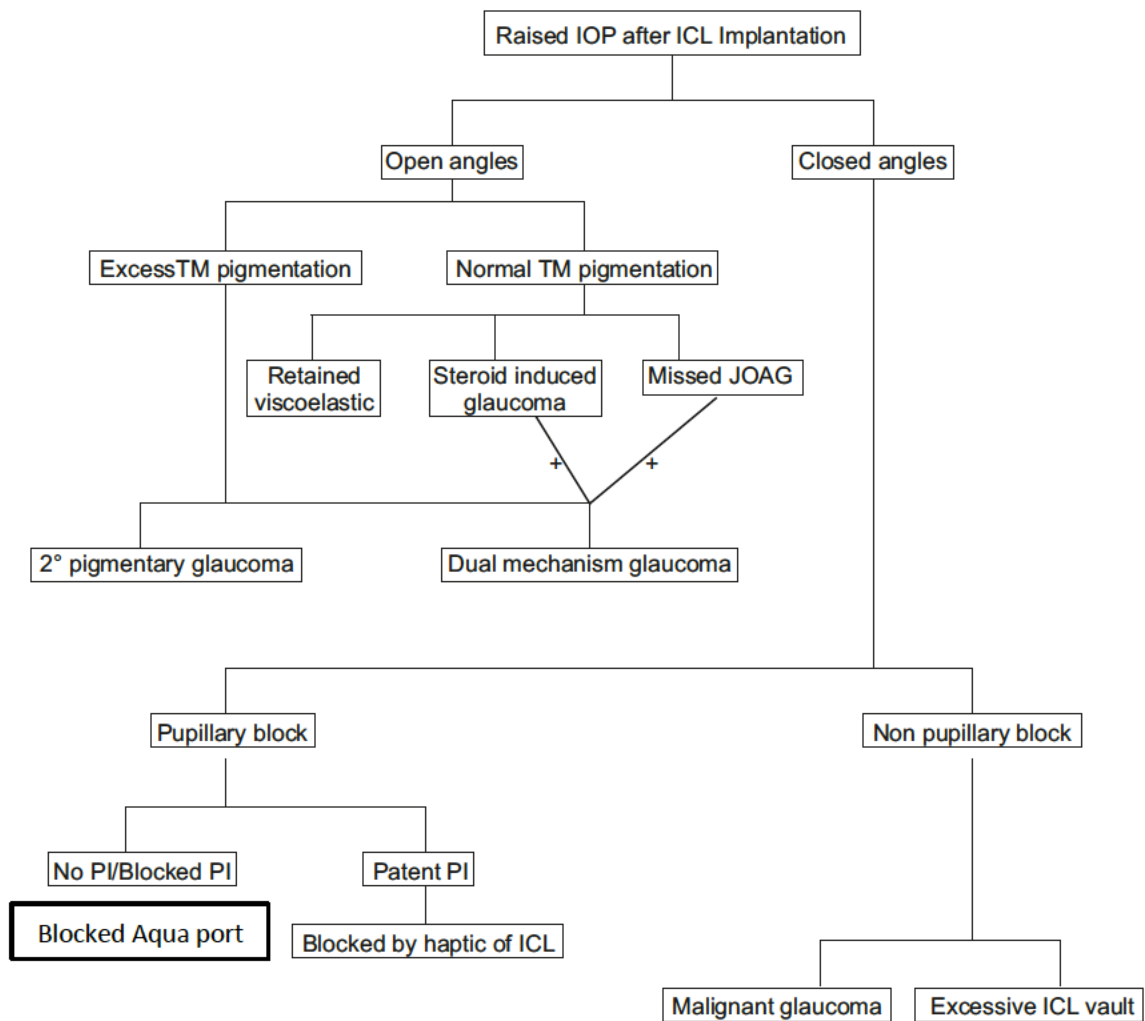

Supplement: S1 Fig — Legends: ICL: Implantable collamer lens, IOP: Intraocular pressure, JOAG: Juvenile open angle glaucoma, PI: Peripheral iridotomy, TM: Trabecular meshwork. (PDF) [file pone.0165469.s001.pdf]
